# Development of medication-related counselling skills in senior medical students: a checklist-based approach

**DOI:** 10.1186/s12909-019-1773-x

**Published:** 2019-09-05

**Authors:** Shalini Gupta, James Shaw

**Affiliations:** Ninewells Hospital and Medical School, University of Dundee, Dundee, DD1 9SY UK

**Keywords:** Effective communication, Patient safety, Medication-related counselling, Undergraduate teaching, Checklist

## Abstract

**Background:**

Effective communication between healthcare providers and patients has been established as a vital element in medication compliance and patient safety. Medical curricula worldwide include medication-related counselling skill as a learning outcome for medical graduates. However, this aspect of health-care training is frequently informal and poorly structured in most medical schools. This paper provides an interesting view of students’ experiences of using a checklist-based approach to develop and practice patient counselling in relation to prescribed medications.

**Methods:**

The authors describe introduction of a thirteen item “Patient Education Checklist” (PEC) as part of an optional checklist based exercise (CBE) in year 4 and 5 clinical blocks. Students consulted PEC to discuss relevant practical issues related to medication intake with their patients. Students were expected to submit reflective case summaries regarding their experience of using PEC to counsel patients over a two-week period. The textual data from student submissions was analysed using inductive content analysis.

**Results:**

We received 13 year4 and 17 year5 student submissions. A content analysis of student reflections identified four dominant themes 1.Enhancement in self-confidence in relation to patient education (86.7%), 2. PEC perceived useful for patient counselling (83.3%), 3. Recognising variation in health literacy levels of patients (50%), 4.Fear of overloading the patient with information (23.3%). Students realised the need to present the medication related knowledge in simple language and tailor the amount of information as per patients’ understanding. Student reflections included interesting observations about the wide variation in health literacy of patients and insights into patients’ concerns and frequent misconceptions about medicines.

**Conclusion:**

Students perceived PEC as a useful tool in adding focus and structure to student patient interactions. They report that it substantially improved their confidence and added quality to patient encounters. Future research is required to assess the effect of CBE on medication compliance and therapeutic outcome. PEC might serve as a useful resource for pharmacy and nursing students.

## Background

National Institute for Health and Clinical Excellence [1] estimates that one-third to one-half of all medicines prescribed for long-term conditions are not taken as recommended. This leads to suboptimal therapeutic benefits as well as raises the risk of adverse drug reactions (ADRs). There are multitudes of underlying reasons for this such as, patients’ inability to comprehend instructions provided by health-care staff, fear of side effects, perceived lack of efficacy and several misconceptions regarding medications [2]. There is a risk of inadequate benefit from medications when patients are not informed of the practical details pertaining to drug intake.

While the problem is multi-factorial, it is likely that interventions targeted to improve doctor-patient communication will increase medication compliance. Effective communication between healthcare providers and patients has been established as a vital element in enhancing medication compliance and patient safety consistently [3, 4]. This paper describes a curricular intervention undertaken in a UK medical school to foster medication related counselling abilities in undergraduate medical students.

Medical curricula worldwide include medication-related counselling skill as a learning outcome for medical graduates [5, 6]. Despite some schools affording practical opportunities for students to interact with patients, there are concerns regarding this aspect of prescribing competency of junior doctors. Wiernik [7] warned of the dangers of declining pharmacology education in medical and nursing schools leading to ill prepared professionals. Even as medical schools and teaching hospitals recognise the importance of fostering medication-related counselling skills in their students, they often fall short of providing adequate training to develop this professional skill [8]. There is limited progress in specific drug-related counselling abilities, possibly due to few available learning resources targeting undergraduate students.

Our medical school prescribing curriculum is guided by Tomorrow’s Doctors’ outcomes [9]. Traditionally, the undergraduate teaching on medication-related counselling has been informal and somewhat unstructured in the course. A recent case of medication-induced severe sunburn in a patient discharged from the hospital without adequate advice on sun-protection, reinforced requirement for a more structured teaching in this area. As a consequence, a checklist based exercise (CBE) to foster medication-related counselling skills of undergraduate students was developed and piloted with senior medical students. Although the World Health Organisation [10] WHO’s Good prescribing guide has questions regarding drug-related information to be conveyed to the patient, we are not aware of any published checklist in the medical literature targeted towards training medical students for this purpose. The authors discuss some of the successes and challenges of this checklist-based approach below.

## Methods

After a series of consultations with pharmacology tutors, clinicians (hospital and primary care physicians), curriculum convenor, teaching leads, students and pharmacists, it was agreed to introduce structured opportunities for student patient interactions with a focus on medication related counselling. In order to ensure that adequate information and instructions regarding prescribed medications are included, and none of the valuable elements are missed during student patient encounters, a need for a comprehensive checklist emphasising each of the criteria was perceived. Various items of drug information were debated upon to get a balance between keeping the checklist concise and simultaneously include all relevant criteria. Eventually, a thirteen-item “**Patient Education Checklist” (PEC**) was developed with consensus from clinicians, pharmacists and pharmacology tutors (Table 1). PEC ensured that students discuss relevant information regarding safety and efficacy of prescribed drugs as well as practical issues in relation to medication intake with their patients.

**Table 1:** Patient Education Checklist (PEC)

Purpose of the prescribed medication/medications	
Frequency & Duration of intake	
Onset of action & Relevance of compliance	
Relation to time (eg. meal times, bedtime)	
Potential ADRs (including phototoxicity, teratogenic actions etc) Any action required (eg. Stop med/report) *Exercise caution regarding the level of details so as not to scare the patient from taking the drug altogether*.	
Dietary restrictions or alcohol limitation/abstinence required	
Drug Interactions (including herbals/OTC products; particularly relevant in polypharmacy)	
Driving restriction required	
Administration Technique optimised/demonstrated	
Consequences of missed doses/abrupt discontinuation	
Lab investigations required/ scheduled	
Non-pharmacological/lifestyle advice (if needed)	
Check with patient if they have any questions/ issues regarding their medicines	

PEC was introduced as an optional activity and as a component of student workbook in the Year 4 “Ageing and Health” and Year 5 “Student Assistantship” blocks. The years 4 and 5 constitute the senior and clinical years of our 5-year spiral curriculum of the undergraduate medical course. These blocks were selected on the basis of relevance of subject and stage of training, practical opportunities and supportive attitude of the clinical team towards student-patient encounters. During the pilot period, 48 Year 4 students doing the “Ageing and Health” block and 69 Year 5 students on the “Student Assistantship” block were offered the opportunity to undertake the CBE.

A period of 2 weeks was assigned to students to do the preparatory study using PEC, counsel real patients and submit completed workbooks. The student workbook comprised of specific learning outcomes inline with Tomorrow’s doctors [9] and instructions on how to use PEC for optimal patient encounter. Students were urged to use *British National Formulary* (BNF) as a reference for medication-related information, including precautions, warnings, ADRs and drug interactions. They were also advised to make use of quality education and patient safety tools such as “chunk and check” technique [11] and “teach back” [12] to optimise their interaction with patients and to improve patient receptivity. The former involves fragmenting information in small chunks to counsel patients, while the later requires patients to repeat in their own words what they had just been told, to check if the information understood by the patient is accurate.

The workbook also included instructions for reflective summary as the following open-ended prompt for CBE -

***Record a reflective summary*** *of your patient counselling experience. Please include the following in your summary:*

- *How many patients did you counsel?*
- *Which are the drugs regarding which you provided patients/carers with the necessary information?*
- *What relevant areas did you counsel the patients/carers on?*
- *Reflect on the experience and the learning you take from this exercise.*

The textual data from student submissions was analysed using inductive content analysis [13]. The corresponding author who is a pharmacology tutor with the medical school analysed all the student submissions.

Students had the autonomy in selecting patients and drugs to practise their counselling skills. Clinical tutors and pharmacists checked on their progress during ward rounds and tutorials, and provided support and supervision as required. Since this was an optional activity, conscious efforts were made to sustain motivation during the two-week period and students were encouraged to share positive experience of patient-counselling with peers to boost engagement. It was highlighted to students in the CBE workbook that not all of the criteria listed may be applicable for every drug prescribed, and students were advised to apply their knowledge of basic pharmacology and exercise judgement to avoid overwhelming the patients with excessive information.

Formal ethical approval was not deemed necessary for this curricular intervention since an audit of standard practice which does not involve identifiable records does not require ethical approval as per the specified criteria in Checklist1 (section A) of the University of Dundee’s *Ethical Approval for Non-clinical research involving human participants* [14].

## Results

The medical school office received 30 student submissions over the 2 month pilot, 13 of which were Year 4 (participation rate 27.1%) and 17 were Year 5 (participation rate 24.6%). All submissions appreciated and rated the CBE as useful and relevant to their stage of training. While most students counselled only one patient, some did take the opportunity to interact with more than one patient. Students engaged in self-learning related toprescribed medications and applied pharmacology before, during and after patient encounters. An inductive content analysis of student reflective essays generated the following dominant themes (Table 2). This paper is based on a curricular intervention (CBE), and the results are generated from a scholarly audit of teaching activity which is exempted from formal ethical approval. However, this limits the authors from sharing the actual student quotes supporting the individual themes.

**Table 2:** Key themes from student submissions

	Emergent Themes	Year 4 (*n* = 13)	Year 5 (*n* = 17)	Total (*n* = 30)
1	Enhancement in self-confidence in relation to patient education after CBE	12 (92.3%)	14 (82.4%)	26 (86.7%)
2	PEC perceived useful for patient counselling	10 (76.9%)	15 (88.2%)	25 (83.3%)
3	Recognising variation in health literacy levels of patients	8 (61.5%)	7 (41.2%)	15 (50%)
4	Fear of overloading the patient with information	4 (30.8%)	3 (17.6%)	7 (23.3%)

### Enhancement in self-confidence in relation to patient education (86.7%)

Most students reported that they felt more confident in their pharmacology knowledge and in the professional skill of counselling patients after the CBE. Year 4 students also valued the opportunity to use BNF and reported feeling more confident and competent navigating around this useful resource. According to students CBE required extensive BNF consultation and that improved their confidence while counselling patients. Year 5 students suggested that introduction of PEC in their previous primary care posting would have led to a more meaningful conversation with patients. Students reported that they have relatively higher patient contact in general practice (GP) block and more independent counselling opportunities on a wider range of medications. It is noteworthy that six out of eleven Year 5 students chose to counsel more than one patient using PEC and reported that they developed confidence with each practice.

### PEC perceived useful for patient counselling (83.3%)

According to year 4 and 5 students who used PEC to prepare for their counselling encounters with patients, the checklist helped to cement their pharmacological knowledge in a setting of clinical realism. One final year student labelled PEC as a “pocketsize tool” to ensure that none of the essential elements are missed during patient encounter. Students commented that PEC prompted them to access the BNF for potential side-effects, drug-interactions and any laboratory monitoring required with specific medications prescribed for their chosen patients. It reminded the students to warn their patient regarding driving restrictions, risks associated with abrupt discontinuations and advice regarding alcohol abstinence when necessary.

### Recognising variation in health literacy levels of patients (50%)

Students made some interesting observations about the wide variation in health literacy of patients. Students reported that some patients were very aware and interested in every possible detail related to their medication and actively asked questions. At the same time there were instances when students perceived their patients to be very ignorant about their disease and drugs. Students who counselled several patients using PEC were in a better position to compare and comment on this variation in health literacy. CBE was perceived as a helpful activity to develop insight into patients’ concerns and frequent misconceptions about medicines.

### Fear of overloading the patient with information (23.3%)

It is noteworthy that nearly a quarter of students who undertook the CBE expressed concerns regarding overwhelming the patients with too much information. This fear was expressed significantly more in the year 4 submissions as compared to Year 5 (30.8% vs 17.6%). Students realised the need to present the medication-related knowledge in simple language and avoid medical terminologies and jargons. Students also reflected on the need to tailor the amount of information to be imparted as per patient’s understanding and relevance. Three year 4 and five year 5 students reported successfully combining “chunk and check” technique along with PEC to avoid burdening their patients with excessive drug-related data.

## Discussion

The overcrowded medical curricula worldwide have been frequently criticised for overburdening the students. Pharmacology is in particular a fact based subject and students often struggle to cope with the vast number of drugs and the related information. Content overload in undergraduate curricula and proliferation of new drugs have been recognized as major contributing factors towards medication errors [15]. Both the British Pharmacological Society guidance for good prescribing [16] and Tomorrow’s Doctors outcomes [9] include “patient-education on the prescribed medications” as an integral part of the prescribing role of a practitioner. It was aimed to simplify the practice of patient counselling through the introduction of PEC which could serve as an educational resource for undergraduate students.

This curricular intervention had a modest aim of exploring the potential of CBE in supporting senior medical students during their patient encounters. The results presented above are derived from student perceptions of their experience of using PEC for patient counselling sessions. Student reflections demonstrate that they viewed PEC as a useful prompt to remind patients of relevant ADRs, drug-interactions, precautions and laboratory monitoring. It served to translate pharmacological knowledge to patient care and was perceived effective in developing confidence in relation to counselling abilities. However, students also expressed concerns regarding overwhelming patients with excessive or unnecessary information. This is understandable given the number of items in the checklist and also the volume of information available in BNF and other relevant resources. We suggest that it might be reasonable to restrict the use of PEC to senior medical students, as in this study. These students already possess background pharmacological knowledge in relation to frequently prescribed drugs and are in a better position to prioritise information in context of individual patients. Students should develop the ability to realistically judge the health literacy level of individual patient with practice, and accordingly tailor the amount of information to impart [11]. PEC should aid in preparation of senior students for their upcoming clinical practice, eventually improving patient compliance, efficiency of drug treatment and clinical outcomes. Similar to any other aspect of the curriculum, medication counselling training too requires time and practice. We anticipate that this CBE adds focus and structure to student-patient interactions and affords development of this professional skill.

We acknowledge that our projections are entirely based on student perceptions and reflections, which may be unreliable. It has not been assessed whether the reported improvement in student confidence also translates into improved clinical practice. Future studies may compare students who receive the intervention with a control group who do not, and track if any positive effect persists over time. According to literature [3], 40–80% medical information given to patients is forgotten immediately and half retained is incorrect. It will be certainly interesting to study the effect of CBE on proper medication use and compliance; although this will require longer term and resource intensive, possibly multi-centric studies. Another significant limitation of the study is that it was introduced as an optional exercise, with the student participation rate being only 27.1 and 24.6% in year 4 and 5 cohorts respectively. It may be argued that the students who undertook the CBE are internally motivated and those who did not volunteer are probably the ones who needed the exercise the most. It is also to be noted that due to limited resources, all the student submissions were analysed by only one researcher, the corresponding author. Analysis by several researchers or peer scrutiny would provide investigator triangulation and improve rigour.

A number of health professionals were involved in drafting PEC and student views were also solicited. However, patients were not consulted. Bleakley and Bligh [17] advocate the potential of patients to play a key role in student learning. We wonder if patients’ perspectives would add value to this intervention resulting in collaborative knowledge production. In addition to medical students, nursing and pharmacy students are also expected to counsel patients on specific aspects of their therapy. It would be worth exploring their perspectives on PEC-based exercise in their practice. PEC has potential to be utilised in inter-professional learning sessions for pharmacy and medical students. Such sessions could improve knowledge and skills of pharmacology and pharmacotherapy for both groups of students as reported in a cross-sectional study [18]. This Dutch study demonstrated that pharmacy students possessed better basic pharmacology knowledge while medical students had better prescription writing skills. Since the CBE requires both basic pharmacological knowledge as well as applied pharmacological principles, the joint interdisciplinary educational workshops should provide opportunities for the two professional groups to learn symbiotically from each other.

## Conclusion

The paper describes the experience of implementing a CBE to foster medication-related counselling skill in undergraduate medical students. Students report that PEC adds focus and structure to student-patient interactions and improves their confidence. The CBE helped learners develop an insight into patients’ concerns and frequent misconceptions about medicines. Future researchers may explore its use in context of medical, pharmacy and nursing students. Our findings suggest that PEC could prove to be a useful educational resource in a variety of pedagogical and clinical settings. This curricular intervention was educational and well-received, and has the potential to affect behavioural change leading to better informed patients who are likely to be more compliant to their medications.

## Data Availability

No datasets were generated, but student submissions that were evaluated to help prepare this commentary are available from the authors on reasonable request.

## Abbreviations

ADR: Adverse Drug Reaction
CBE: Checklist Based Exercise
PEC: Patient Education Checklist
